# Investigating the potential neuroprotective benefits of taurine and Dihydrotestosterone and Hydroxyprogesterone levels in SH-SY5Y cells

**DOI:** 10.3389/fnagi.2024.1379431

**Published:** 2024-05-29

**Authors:** Hailah M. Almohaimeed, Amany I. Almars, Fayez Alsulaimani, Ahmed M. Basri, Norah A. Althobaiti, Aishah E. Albalaw, Ifat Alsharif, Waleed Al Abdulmonem, Almonther Abdullah Hershan, Mona H. Soliman

**Affiliations:** ^1^Department of Basic Science, College of Medicine, Princess Nourah bint Abdulrahman University, Riyadh, Saudi Arabia; ^2^Department of Medial Laboratory Sciences, Faculty of Applied Medical Science, King Abdulaziz University, Jeddah, Saudi Arabia; ^3^Biology Department, College of Science and Humanities Al Quwaiiyah, Shaqra University, Al Quwaiiyah, Saudi Arabia; ^4^Department of Biology, Faculty of Science, University of Tabuk, Tabuk, Saudi Arabia; ^5^Department of Biology, Jamoum University College, Umm Al-Qura University, Makkah, Saudi Arabia; ^6^Department of Pathology, College of Medicine, Qassim University, Buraidah, Saudi Arabia; ^7^Department of Medical Microbiology and Parasitology, College of Medicine, The University of Jeddah, Jeddah, Saudi Arabia; ^8^Botany and Microbiology Department, Faculty of Science, Cairo University, Giza, Egypt; ^9^Biology Department, Faculty of Science, Taibah University, Yanbu, Saudi Arabia

**Keywords:** Alzheimer’s disease, taurine, Dihydrotestosterone, Dihydroprogesterone, miRNA-21, miRNA-181

## Abstract

**Background:**

Taurine, an amino acid abundantly found in the brain and other tissues, has potential neuroprotective properties. Alzheimer’s disease (AD) is a commonly occurring type of dementia, which becomes more prevalent as people age. This experiment aimed to assess the neuroprotective effects of taurine on SH-SY5Y cells by examining its impact on Dihydrotestosterone (DHT), Dihydroprogesterone (DHP), as well as the expression of miRNA-21 and miRNA-181.

**Methods:**

The effects of various taurine concentrations (0.25, and 0.75 mg/mL), and LPS (0.1, and 12 mg/mL) on the SH-SY5Y cell line were assessed using the MTT assay. The levels of DHT and DHP were quantified using an ELISA kit. Additionally, the expression levels of miRNA-181 and miRNA-21 genes were examined through Real-Time PCR analysis.

**Results:**

The results of the MTT assay showed that treatment with taurine at concentrations of 0.25, and 0.75 mg/mL reduces the toxicity of LPS in SH-SY5Y cells. ELISA results indicated that taurine at a concentration of 0.25, and 0.75 mg/mL significantly elevated DHT and DHP hormones in the SH-SY5Y cell line compared to the untreated group (*p* < 0.01). The expression levels of IL-1β and IL-6 were decreased under the influence of LPS in SH-SY5Y cells after taurine treatment (p < 0.01). Gene expression analysis revealed that increasing taurine concentration resulted in heightened expression of miRNA-181 and miRNA-21, with the most significant increase observed at a concentration of 0.75 mg/mL (*p* < 0.001).

**Conclusion:**

Our study findings revealed that the expression of miRNA-181 and miRNA-21 can be enhanced by taurine. Consequently, exploring the targeting of taurine, miRNA-181, and miRNA-21 or considering hormone therapy may offer potential therapeutic approaches for treating AD or alleviating severe symptoms. Nonetheless, in order to fully comprehend the precise mechanisms involved, additional research is required.

## 1 Introduction

Alzheimer’s disease (AD) is a prevalent neurodegenerative disorder, with its occurrence rising among the elderly population. It is distinguished by a range of brain abnormalities, notably neuroinflammation, and progressive neuronal loss. Consequently, individuals with AD commonly exhibit cognitive decline and memory problems (Umoh et al., 2024). Although the exact pathogenesis of AD remains unclear, but the prevailing hypothesis suggests that the disease is initiated and driven by an increased accumulation of Aβ. As a result, there is currently no conclusive cure for AD. However, patients are typically provided with symptomatic treatment, which aims to alleviate their symptoms. It is important to note that while symptomatic treatment does not halt the progression of the disease, it does help to slow it down (DeTure and Dickson, 2019). Research indicates that reduced levels of estrogen and progesterone in women may elevate the susceptibility to AD, whereas a decline in testosterone production as men age may also heighten the risk of AD (Appiah et al., 2024). Additionally, the extension of menopause and the resulting prolonged exposure to female sex hormones lead to a postponement of cognitive decline in women (Radaghdam et al., 2021). Targeting steroid hormones as a means to mitigate the severity of AD holds potential benefits.

MicroRNAs (miRNAs) are small RNA molecules that control gene expression by suppressing or degrading messenger RNAs (mRNA) that are involved in protein production (Hernández-Contreras et al., 2024). Research indicates that the majority of coding proteins are influenced by miRNAs. This means that a single miRNA can govern multiple genes, and conversely, multiple miRNAs can collectively impact a single gene. Consequently, a particular miRNA possesses the ability to regulate diverse cellular processes, including apoptosis, growth, and cell proliferation (Letafati et al., 2022; Shademan et al., 2023). Although miRNA-21 is nonspecificity, it is regarded as a versatile regulator in the advancement of CNS disorders. It is believed to have both harmful and beneficial effects (Garcia et al., 2022; Yang et al., 2022; Huang et al., 2024). In both *in vitro* and *in vivo*, miRNA-21 has been linked to the control of Aβ oligomer-induced toxicity (Xie et al., 2019). Down-regulation of miRNA-181 has been demonstrated in the cerebrospinal fluid of individuals diagnosed with AD (Abdel Gaber et al., 2023). Notably, miRNA-181 demonstrates an inverse correlation with the levels of IL-6 and TNF-a but demonstrates a positive association with anti-inflammatory cytokines like TGF-b and IL-10. This suggests that microRNAs found in the bloodstream have the potential to be used as indicators for the aging process (Indrieri et al., 2020). Furthermore, the absence of this specific miRNA leads to elevated levels of serine palmitoyl transferase (SPT), thereby causing an increase in Aβ levels (Miya Shaik et al., 2018). The significance of miRNA-21 and miRNA-181 in AD is highlighted by this data, indicating their potential as therapeutic targets for treating the disease.

Taurine, also known as 2-aminoethanesulfonic acid, holds the distinction of being the second most prevalent naturally occurring amino acid in the central nervous system, right after glutamate. This versatile compound serves a multitude of functions within the body, encompassing activities such as regulating body temperature, ensuring the proper folding of proteins, combatting inflammation, countering oxidative stress, maintaining osmotic balance, preserving calcium equilibrium, and contributing to the development of the central nervous system (Geekiyanage and Chan, 2011). Prior studies in the scientific literature have indicated a deficiency of taurine in the brains of individuals diagnosed with AD (Oh et al., 2020). Taurine has exhibited promising therapeutic potential in the treatment of neurological disorders, including AD (Rafiee et al., 2022). The research findings indicate that taurine exhibits a modest ability to bind to Aβ plaques, resulting in a limited anti-fibrillogenic impact. Furthermore, when taurine is administered intravenously, it effectively mitigates Aβ neurotoxicity and cognitive impairment (Santa-María et al., 2007; Jakaria et al., 2019). As of now, there have been no documented reports of potential side effects associated with taurine. Given its non-toxic nature within the body, taurine has found application in various food products (Geekiyanage and Chan, 2011). In this research, we delved into the impact of taurine on the toxicity of SH-SY5Y cells. Moreover, we quantified alterations in DHT and DHP concentrations in the presence of taurine. Additionally, we assessed variations in the expression of miRNA-21 and miRNA-181 within SH-SY5Y cells exposed to taurine. The findings from this investigation may provide insights into potential therapeutic avenues for individuals with AD.

## 2 Material methods

### 2.1 Cell line, chemicals and culture conditions

In our research, we assessed the protective impact of taurine on the SH-SY5Y cell line through cell culture techniques. The SH-SY5Y cells used in this study were obtained from the American Type Culture Collection and cultured according to standard procedures. Cell lines were cultured in DMEM/HG medium (Biological Industries) supplemented with 10% fetal bovine serum (FBS) (Biological Industries), 2 mM L-Glutamine (Biological Industries), and 1% penicillin–streptomycin (Biological Industries). The cells were cultured for 24 h in a controlled environment at 37°C, with a humidity level of 95 and 5% CO2.

### 2.2 Treatment of SH-SY5Y cells with lipopolysaccharides and taurine

Due to the expression of Aβ, tau, inflammatory factors, and other neuron-specific proteins, SH-SY5Y cells serve as a suitable model to investigate the mechanism of neuron phenotype degeneration, including AD. To differentiate SH-SY5Y cells, we treated them with different concentrations of lipopolysaccharide (LPS) (0.1, 12 mg/mL). Following this incubation period, the cells were exposed to varying concentrations of taurine (0.25, and 0.75 mg/mL) (Rossato et al., 2021) for an additional 24 h. Also, to check the differentiation of these cells, we checked the expression of some pro-inflammatory (IL-6, and IL-1β) cytokines after 12, and 24 h of treating the cells with lipopolysaccharides and taurine.

### 2.3 Measurement of cytotoxicity

To assess the individual and combination impacts of taurine and LPS toxicity on SH-SY5Y cells, we employed the MTT method, conducting three replicates over a single day. We initiated the experiment by seeding SH-SY5Y cells at a density of 5 × 10^4^ cells per well in 96-well plates. These cells were then exposed to various taurine concentrations (0.25, and 0.75 mg/mL), and LPS (0.1, and 12 mg/mL) for 24 h. To gauge SH-SY5Y cell viability, we employed the MTT assay provided by Gibco (United States). The protocol entailed replacing the old medium with a fresh medium containing an MTT solution (5 mg/mL reagent in PBS), followed by a 4-h incubation under standard conditions. After this incubation, 50 μL of dimethyl sulfoxide (DMSO) was introduced into each well, and a 30-min incubation was performed to halt the reaction. Quantitative measurement of the formazan dye produced during this process was executed on a microplate reader (Multiskan FC, Thermo) at 450 nm absorbance with a 620 nm reference range, taking measurements every 15 min.

### 2.4 Examining changes in DHT and DHP levels

My BioSource kits (Cat. No: MBS762135) were utilized to assess the levels of Dihydrotestosterone (DHT) and Hydroxyprogesterone (DHP). To achieve this, SH–SY5Y cells were initially seeded at a density of 5 × 10^4^ cells per well in 96-well plates, followed by the implementation of the designated treatment protocol. To remove insoluble impurities and cellular debris, the supernatant from the cell culture was subjected to centrifugation at 1000 × g for 20 min at 2–8°C. Subsequently, the supernatant was diluted in a 1:2 ratio using the supplied buffer that was supplemented with a protease inhibitor to prevent protein degradation. Standards and samples were prepared following the instructions provided with the kits. The optical density of the samples was then measured using a microplate reader (Thermo Multiskan FC) at a wavelength of 450 nm.

### 2.5 Evaluation of the effects of taurine and LPS on IL-6 and IL-1β in the SH–SY5Y cells

The SH–SY5Y cells were initially distributed into a 6-well plate at a density of 5 × 10^4^ cells per well and cultured under standard conditions for 24 h. Subsequently, these SH–SY5Y cells were subjected to various taurine concentrations (0.25, and 0.75 mg/mL) and LPS (0.1, and 12 mg/mL) for another 24-h period. Total RNA was extracted from both the taurine-treated SH–SY5Y cells and the untreated control cells using TRIzol reagent (Invitrogen; Thermo Fisher Scientific, Inc.), following the guidelines provided by the manufacturer (Invitrogen, United States). Ultimately, the volume of the solution was augmented to 10 μL by introducing 3 μL of Diethyl pyrocarbonate (DEPC) water. The extracted RNA’s quality and quantity were subsequently assessed using electrophoresis on a 1% agarose gel and a NanoDrop instrument (Thermo Scientific, USA). Next, cDNA synthesis was conducted in accordance with the manufacturer’s guidelines provided by PrimeScript™RT Reagent kit with Genomic DNA Eraser (Takara Biotechnology Co., Ltd.). qPCR was performed on a Chromo4 Four-Color Real-Time PCR Detection system (Bio-Rad Laboratories, Inc., Hercules, CA, USA) using the SYBR® Premix Ex Taq™II (Tli RNaseH Plus) kit (Takara Biotechnology Co., Ltd.). The PCR protocol followed this sequence: initial denaturation (1 cycle at 95°C for 2 min), denaturation (40 cycles at 95°C for 30 s), annealing (40 cycles at 60°C for 20 s), extension (40 cycles at 72°C for 20 s), and a final extension step (1 cycle at 72°C for 5 min). The β-actin gene served as the endogenous control. Primers were synthesized by Thermo Fisher Scientific, Inc. (Table 1).

**Table 1:** Primer sequences.

Gene Name	Primer Sequence
*miRNA-21*	Forward: 5′-GCCCGCTAGCTTATCAGACTGATG-3′ Universal Reverse: 5′-CAGTGCAGGGTCC GAGGT-3′
*miRNA-181*	Forward: 5′-CAGTGAACATTCAACGCTGTC-3′ Universal Reverse: 5′-GCTGATGGTTGGCCATAGG-3′
*IL-6*	Forward: 5′-AGAGACTTCCAGCCAGTTGC-3′ Universal Reverse: 5′-AGTCTCCTCTCCGGACTTGT-3′
*IL-1β*	Forward: 5′- CCTTGTCGAGAATGGGCAGT-3′ Universal Reverse: 5′-TTCTGTCGACAATGCTGCCT-3′
*β-actin*	Forward: 5′-AGAGCTACGAGCTGCCTGAC-3′ Universal Reverse: 5′-AGCACTGTGTTGGCGTACAG-3′

### 2.6 Evaluation of the effects of taurine and LPS on miRNA-181 and miRNA-21 gene expression levels in the SH–SY5Y cells

The SH–SY5Y cells were initially distributed into a 6-well plate at a density of 5 × 10^4^ cells per well and cultured under standard conditions for 24 h. Subsequently, these SH–SY5Y cells were subjected to various taurine concentrations (0.25, and 0.75 mg/mL) and LPS (0.1, and 12 mg/mL) for another 24-h period. Total RNA was extracted from both the taurine-treated SH–SY5Y cells and the untreated control cells using TRIzol reagent (Invitrogen; Thermo Fisher Scientific, Inc.), following the guidelines provided by the manufacturer (Invitrogen, United States). Ultimately, the volume of the solution was augmented to 10 μL by introducing 3 μL of Diethyl pyrocarbonate (DEPC) water. The extracted RNA’s quality and quantity were subsequently assessed using electrophoresis on a 1% agarose gel and a NanoDrop instrument (Thermo Scientific, United States). Next, cDNA synthesis was conducted in accordance with the manufacturer’s guidelines provided by PrimeScript™RT Reagent kit with Genomic DNA Eraser (Takara Biotechnology Co., Ltd.). The sequences of the miRNA-21 and miRNA-181 primers can be found in Table 1. The changes in mRNA expression of miRNA-21, and miRNA-181 genes were evaluated using quantitative real-time PCR (qRT-PCR) (Bio Rad, United States). The PCR reaction was conducted in a total volume of 10 μL, consisting of 5 μL of PCR pre-Mix, 1 μL of cDNA, 0.5 μL of forward primer, and 0.5 μL of reverse primers. The PCR protocol followed this sequence: initial denaturation (1 cycle at 94°C for 3 min), denaturation (40 cycles at 94°C for 15 s), annealing (40 cycles at 60°C for 20 s), extension (40 cycles at 72°C for 25 s), and a final extension step (1 cycle at 72°C for 5 min). The β-actin gene served as the endogenous control.

### 2.7 Statistical analysis

The study’s experiments were conducted in triplicate, and the resulting data, derived from three distinct experiments, were depicted as the mean value along with the corresponding standard deviation (SD). Statistical analysis involved the application of one-way analysis of variance (ANOVA), followed by Tukey’s *post hoc* test, and conducted using GraphPad Prism (version 8.4.2). For gene expression, data was normalized using the 2^-ΔΔCq^ method. Significance was attributed to *p*-values less than 0.05.

## 3 Results

### 3.1 Expression level of IL-6 and IL-1β

**Figure 1:**
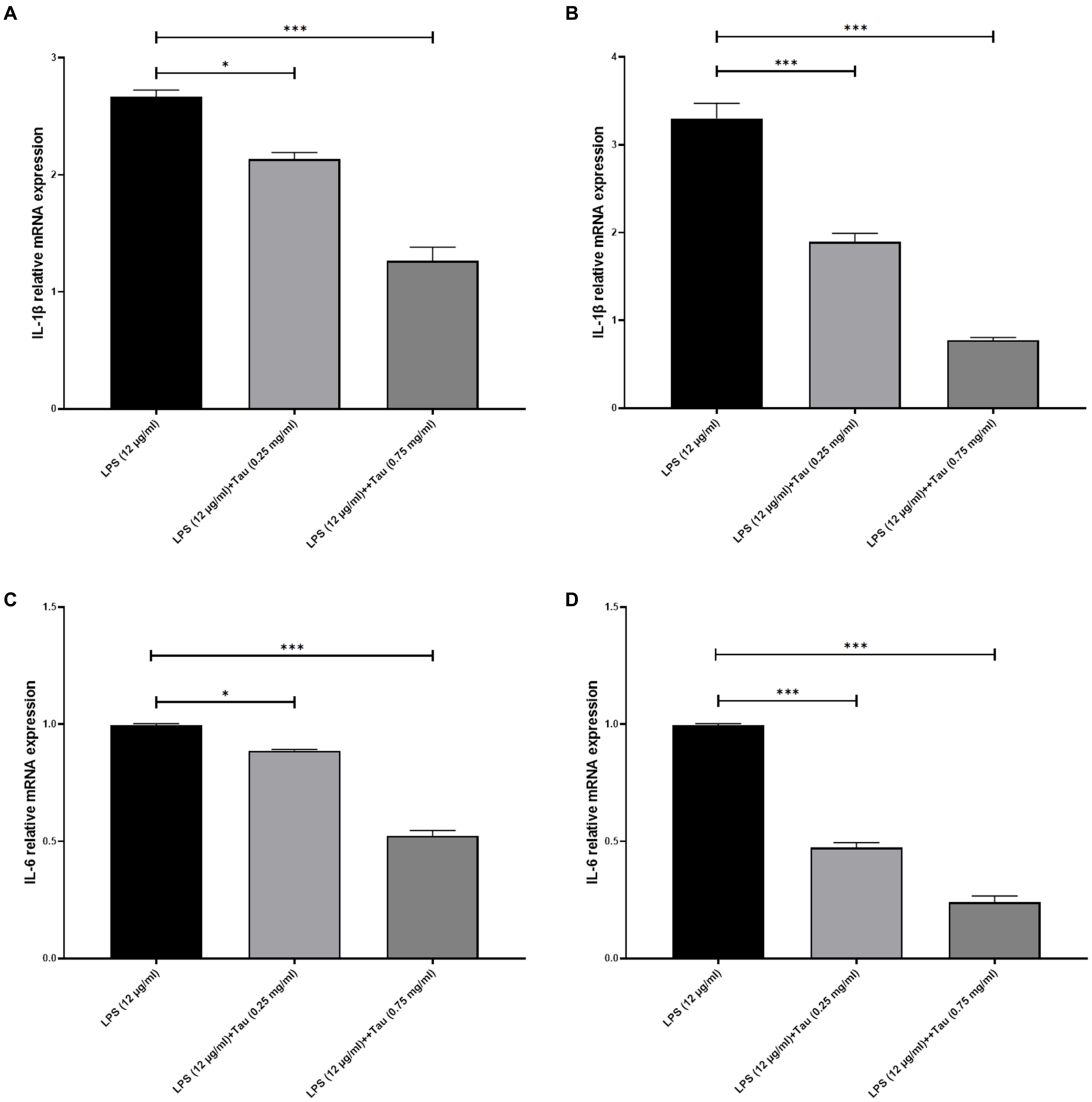
Taurine reduces the concentration of IL-1β and IL-6 in SH-SY5Y cells. RT-qPCR analysis of IL-1β following **(A)** 12, **(B)** 24 h of Taurine treatment. RT-qPCR analysis of IL-6 following **(C)** 12, **(D)** 24 h of Taurine treatment. Data are presented as fold-change vs. the LPS alone group (^*^p < 0.05, ^**^p < 0.01 and ^***^p < 0.001). IL, interleukin; LPS, lipopolysaccharide; Tau, Taurine.

Total RNA was prepared from SH-SY5Y cells pretreated with LPS (0.1, and 12 μg/mL) for 24 h and exposed to Taurine (0.25 and 0.75 mg/mL) for 12, and 24 h. The expression level of IL-1β and IL-6 under the influence of LPS (12 μg/mL) in SH-SY5Y cells decreases after treatment with Taurine (Figure 1). Taurine shows more effective effects after 24 h than after 12 h (*p* < 0.001).

### 3.2 Evaluation of the effects of taurine and LPS on SH–SY5Y cells

**Figure 2:**
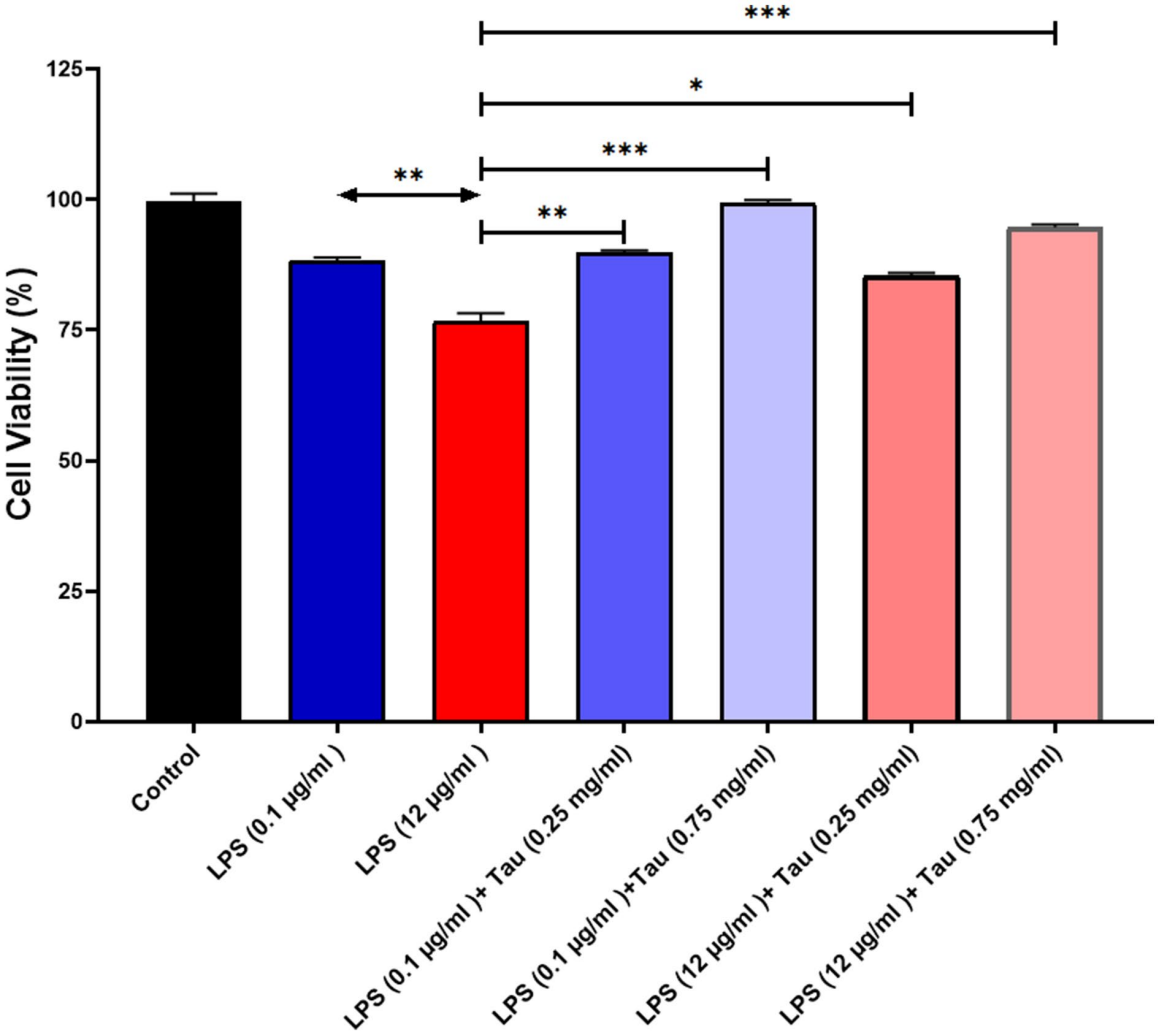
SH-SY5Y viability on different concentrations of taurine concentrations (0.25, and 0.75 mg/mL) and LPS (0.1, and 12 mg/mL) compared to the control group.

In order to assess the responsiveness of SH-SY5Y cells to taurine, we conducted a 24-h experiment where cell viability was evaluated across a diverse range of taurine concentrations (0.25, and 0.75 mg/mL) and LPS (0.1, and 12 mg/mL). The results of the study showed that treatment with taurine at concentrations of 0.25, 0.75 mg/mL reduces the toxicity of LPS in SH-SY5Y cells (Figure 2).

### 3.3 Evaluation of the effects of taurine and LPS on DHT and DHP levels

**Figure 3:**
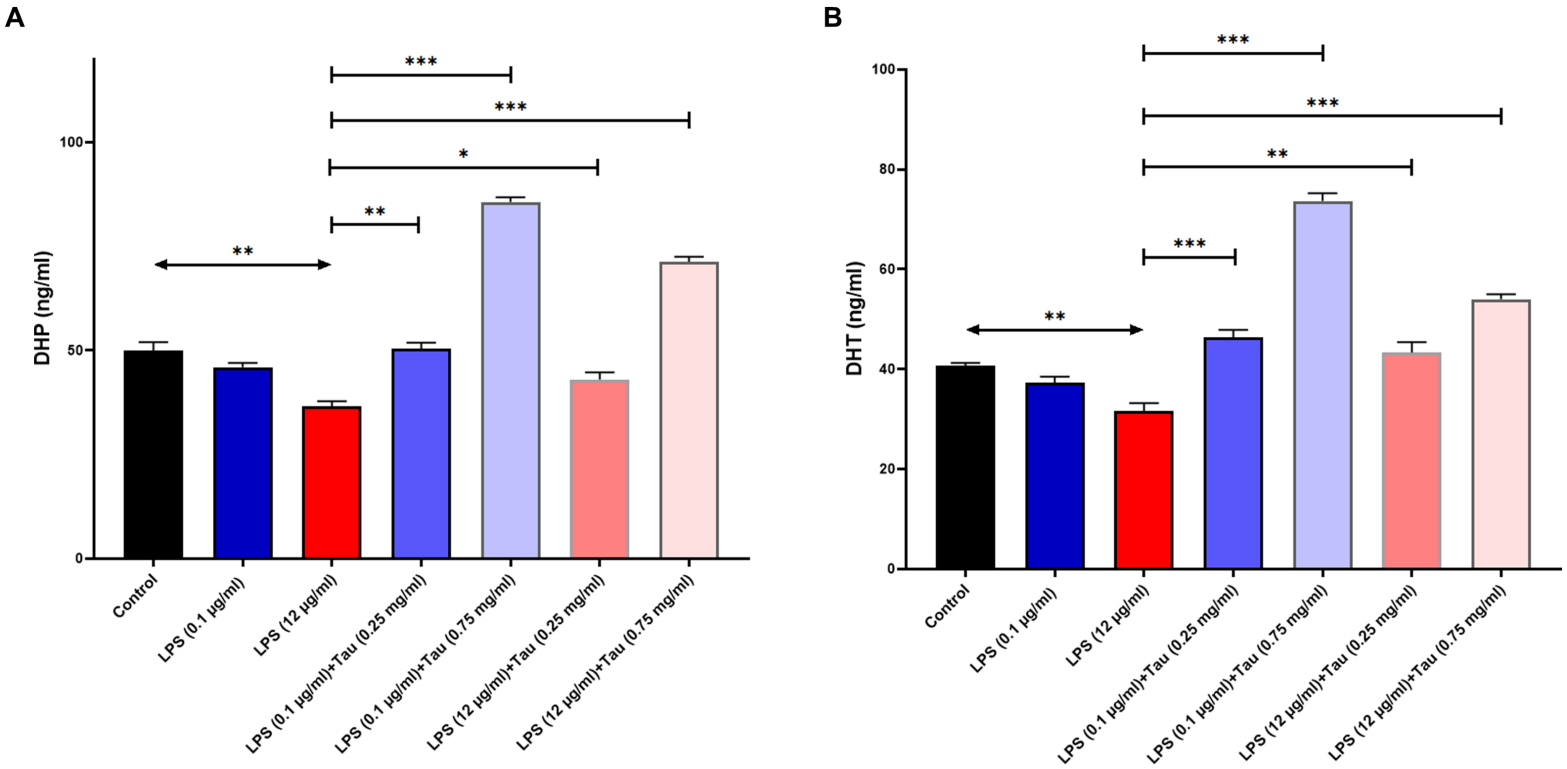
Taurine increases the concentration of **(A)** Dihydroprogesterone and **(B)** Dihydrotestosterone in the SH-SY5Y cells (^*^p < 0.05, ^**^p < 0.01 and ^***^p < 0.001).

ELISA results showed that taurine at a concentration of 1 mg/mL, compared to the untreated group, significantly increased DHT and DHP hormones in the SH-SY5Y cell line (p < 0.001). The lowest increase in the concentration of DHT and DHP in SH-SY5Y cells was related to the cells treated with taurine at a concentration of 0.1 mg/mL (*p* < 0.05) (Figure 3). Although the low concentration of taurine did not have a noticeable effect on the increase of DHT and DHP hormones, with the increase of taurine concentration, the increase of DHT and DHP hormones was significant.

### 3.4 Determination of gene expression of miRNA-21, and miRNA-181

**Figure 4:**
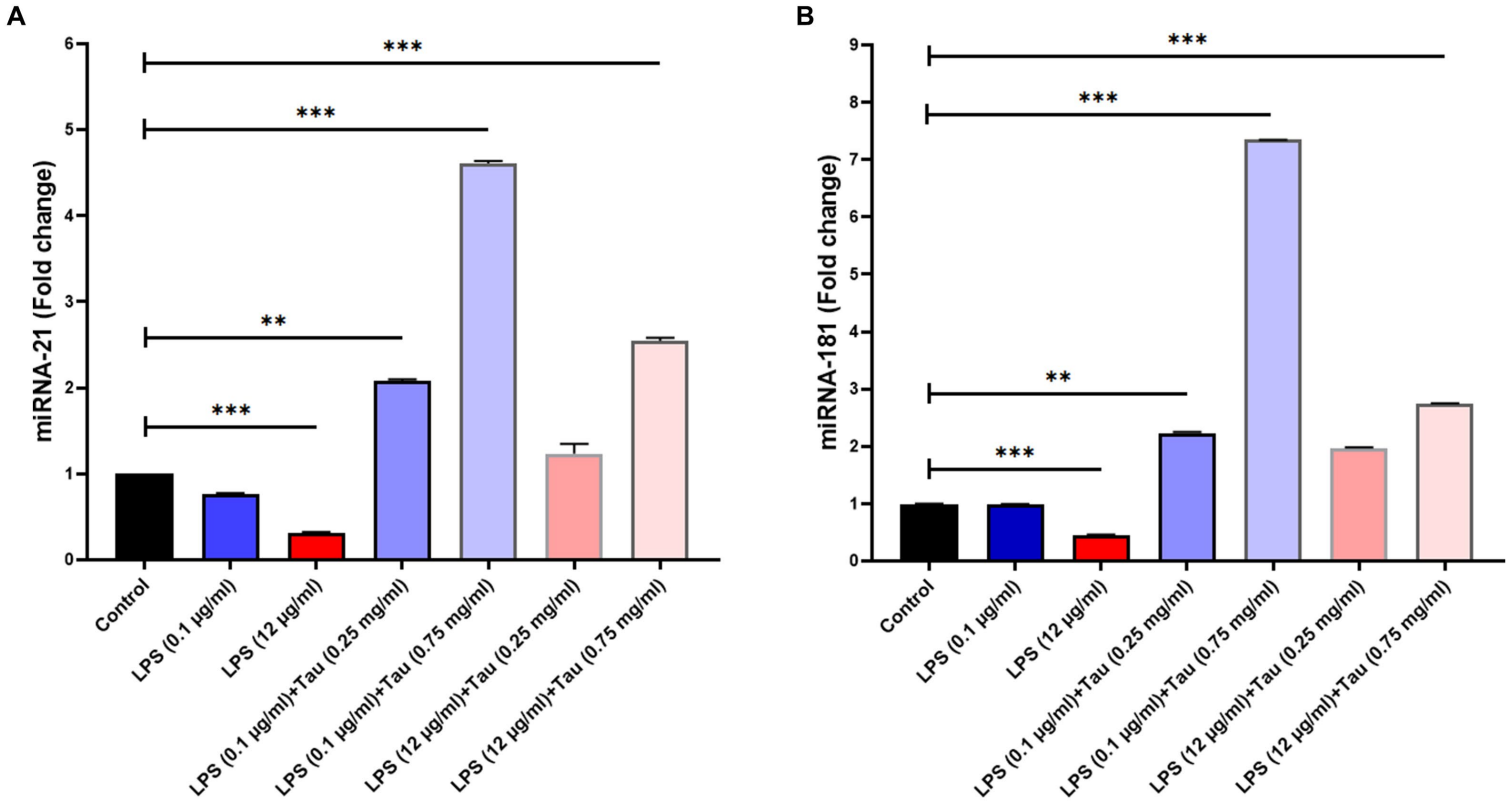
Differences in miR-21 and miR-181 expression between different doses of taurine concentrations (0.25, and 0.75 mg/mL) and LPS (0.1, and 12 mg/mL) in SH-SY5Y cells. This figure shows the expression of miR-21 and miR-181 in the SH-SY5Y cell line. **(A)** Rate of change in miR-21 expression between the groups. **(B)** Rate of differences in miR-181 expression between the groups (^**^p < 0.01 and ^***^p < 0.001).

The expression of miRNA-21 was increased following treatment with taurine concentrations (0.25, and 0.75 mg/mL) and LPS (0.1, and 12 mg/mL) in SH-SY5Y cells. Our results showed that the concentration of 0.75 mg/mL of taurine increases the expression of miRNA-21 (*p* < 0.001), while at the concentration of 0.25 mg/mL, it is accompanied by a slight increase in the expression of miRNA-2 (*p* > 0.05) (Figure 4A).

The expression of miRNA-181 genes was increased following treatment with taurine concentrations (0.25, and 0.75 mg/mL) and LPS (0.1, and 12 mg/mL) in SH-SY5Y cells. Our results showed that increasing the concentration of taurine increases the expression of miRNA-181, but the lowest expression of miRNA-181 was observed at a concentration of LPS (12 mg/mL) (*p* < 0.001) (Figure 4B).

## 4 Discussion

In youthful communities, individuals tend to undergo the aging process over the course of several decades. Considering that AD predominantly affects older individuals, it becomes evident that progress in the field of cell biology, aimed at comprehending the intricate molecular mechanisms at the core of AD, is indispensable for effective treatment and disease management. The complexity of AD’s molecular pathogenesis is well-known, encompassing various interconnected hypotheses and a multitude of factors contributing to the disease. Nevertheless, these hypotheses in isolation fall short of providing a comprehensive explanation and interpretation of all facets of AD pathology. Further research is imperative to delve into aspects such as the fundamental origins of the condition, including the aberrant genesis of Aβ and the intricate mechanisms through which it impacts neurons. Additionally, the intricate interplay between aging, gender, and cognitive impairments in AD remains a topic that is not yet fully elucidated.

Since taurine is a naturally occurring amino acid, it is expected to have minimal adverse effects on the body. Toxicity studies have indicated that it does not exhibit genotoxic, carcinogenic, or teratogenic properties (Jakaria et al., 2019; Srivastava et al., 2022). Nevertheless, a few research studies have indicated acceptable taurine limits based on the No Observed Adverse Effect Level (NOAEL) (Cantafora et al., 1986; Duan et al., 2023). Following treatment with specific concentrations of taurine, there was no notable impact on cell viability, suggesting that these concentrations do not pose any toxicity to SH-SY5Y cells. This is especially important because the utilization of specific taurine quantities can yield diverse outcomes, making it crucial to pinpoint these precise amounts for practical insights.

The expression level of IL-1β and IL-6 under the influence of LPS (12 μg/mL) in SH-SY5Y cells decreases after treatment with Taurine. Taurine shows more effective effects after 24 h than after 12 h. SIRT1 and SIRT2 modulators mitigate the inflammatory response induced by lipopolysaccharide (LPS) in HAPI microglial cells and confer protection to SH-SY5Y neuronal cells *in vitro* (Zhang et al., 2021).

The ELISA findings demonstrated a notable elevation in DHT and DHP hormone levels in the SH-SY5Y cell line when treated with taurine at a concentration of 0.75 mg/mL, in comparison to the untreated group. While the low taurine concentration did not produce a discernible impact on the elevation of DHT and DHP hormones, a notable increase in taurine concentration resulted in a significant rise in DHT and DHP hormone levels. The decline in sex hormones like progesterone, estradiol (E2), and testosterone as ages serve as compelling evidence linked to the development of AD pathogenesis (Frontzek et al., 2016). Furthermore, the findings from Verdile’s (Verdile et al., 2014) research demonstrated that testosterone has the ability to lower serum Aβ levels while enhancing its elimination in brain tissue. Reduced levels of testosterone in males are linked to cognitive deterioration and the incidence of AD. In individuals diagnosed with AD and dementia, their testosterone levels are lower compared to those without these conditions who are otherwise healthy (Sundermann et al., 2020). Progesterone, primarily produced in the ovaries, has demonstrated its neuroprotective capabilities in various disease models, including AD, stroke, and traumatic brain injury (Stein et al., 2008; Singh and Su, 2013; Canfrán-Duque et al., 2017). Taurine has been shown to increase Sirtuin 1 gene expression in various cells such as the liver and heart (Feng et al., 2018; Wang et al., 2023). In our experiments, LPS concentration may control DHT and DHP levels by affecting Sirtuin 1 gene expression. These need to be further investigated in future studies. These findings suggest that lower levels of testosterone and progesterone may pose a risk factor for AD and Hormone therapy could be contemplated as a potential approach for managing and treating neurodegenerative conditions, including Alzheimer’s disease.

Our findings indicate that as the concentration of taurine increases, there is an elevation in the expression levels of miRNA-21 and miRNA-181. The lowest expression of miRNA-21 and miRNA-181 was observed when the concentration was set at 0.75 mg/mL. MiRNA-21 has been implicated in numerous disease processes. When miRNA-21 levels are reduced in macrophages, it can trigger apoptosis, promote plaque necrosis, and exacerbate inflammation during the progression of atherosclerosis (Song et al., 2023). Increased levels of miRNA-21 have the potential to impede the cellular apoptosis induced by Aβ1-42. These results indicate that elevating miRNA-21 expression could potentially mitigate SH-SY5Y cell apoptosis, possibly offering a protective role in the development of Alzheimer’s disease (Ghorbani et al., 2017). The miRNA-181 miRNA family comprises a remarkably conserved set of miRNAs that exert influence over diverse facets of cellular biology, encompassing cell proliferation, differentiation, and apoptosis (Indrieri et al., 2020). Additional research uncovered that the reduced expression of miRNA-181 correlated with an elevated Aβ expression level, indicating that miRNA-181 is indeed suppressed in AD (). The interplay between miR-181a-5p and sirtuin 1 plays a crucial role in governing the differentiation and apoptosis of human bone marrow mesenchymal stem cells (Zhu et al., 2021). The increased expression of these microRNAs, observed at a concentration of 0.75 mg/mL of taurine, might be associated with the modulation of Sirtuin 1 gene expression, thereby implicating their potential relevance in neuroprotection. Analyzing the expression levels of particular miRNAs in AD holds potential for advancing our understanding and strategies for treating and managing the condition.

The role of taurine as an activator of Sirtuin 1 has been demonstrated in the literature (Abd Elwahab et al., 2017). Based on the previous data, there is a clear need to research taurine to ascertain its potential side effects in various cellular and animal models (Arya Devi et al., 2022). Investigating the role of Sirtuin 1 after the treatment of cells with lipopolysaccharides and taurine is suggested in future studies.

## 5 Conclusion

In conclusion, our experiment demonstrated the potential neuroprotective effects of taurine on SH-SY5Y cells, a cell line commonly used in neurobiology research. Taurine, at concentrations of 0.25 and 0.75 mg/mL, exhibited a protective effect against the toxicity induced by LPS in SH-SY5Y cells, as evidenced by the MTT assay results. Furthermore, taurine treatment significantly elevated the levels of DHT and DHP hormones, indicating a potential hormonal modulation effect. Notably, the study unveiled a novel aspect of taurine’s impact on miRNA expression, particularly miRNA-181 and miRNA-21. The upregulation of these miRNAs, especially at a concentration of 0.75 mg/mL, suggests a potential regulatory role of taurine in gene expression associated with neuroprotection. The observed decrease in IL-1β and IL-6 expression levels further supports the anti-inflammatory potential of taurine in the context of neurodegeneration. Furthermore, at higher concentrations, taurine significantly heightened the presence of DHT and DHP hormones in the SH-SY5Y cell line when compared to the untreated group. Targeting these molecular pathways may offer promising strategies for intervention or hormone therapy to mitigate severe symptoms associated with AD. However, to fully comprehend the precise mechanisms and validate the translational potential of these observations, additional in-depth research and clinical studies are warranted. Our study lays a foundation for future investigations aimed at unraveling the intricate molecular mechanisms underlying taurine’s neuroprotective effects and its potential application in the development of novel therapeutic approaches for AD.

## Data availability statement

The original contributions presented in the study are included in the article/supplementary material, further inquiries can be directed to the corresponding author.

## Author contributions

HA: Conceptualization, Data curation, Formal analysis, Methodology, Writing – original draft. AmA: Formal analysis, Methodology, Writing – original draft. FA: Data curation, Methodology, Writing – original draft. AB: Methodology, Writing – original draft. NA: Formal analysis, Methodology, Writing – original draft. AiA: Formal analysis, Writing – original draft. IA: Data curation, Formal analysis, Writing – original draft. WA: Data curation, Methodology, Resources, Writing – original draft. AH: Investigation, Validation, Writing – original draft. MS: Conceptualization, Project administration, Validation, Visualization, Writing – original draft, Writing – review & editing.
